# Perception, theory, and the mistaken mind

**DOI:** 10.3389/fnhum.2014.00543

**Published:** 2014-07-24

**Authors:** Rebecca F. Schwarzlose

**Affiliations:** Laboratory for Cognitive and Brain Development, Institute of Gerontology, Wayne State UniversityDetroit, MI, USA

**Keywords:** book review, perception, theory, representation, popular psychology, faces, publishing

What do we know and how do we know it? These are the weighty questions posed by Viki McCabe in her recent book, *Coming to Our Senses: Perceiving Complexity to Avoid Catastrophes*. The book deconstructs disasters ranging from the tragic attack on Iran Air Flight 655 to the rise of Wall Street's derivatives market and the construction of no-outlet levees that would eventually fail New Orleans. In the process, it highlights how decision makers went wrong and draws grand conclusions about the nature of human perception and mental representation.

McCabe's central argument hinges on the idea that valuable clues about natural systems are revealed through structural information, or patterns that characterize everything from the branching tree limb to the spiraling whirlpool. Humans struggle to mentally represent complex systems and the patterns they produce. We try to translate structural information into symbols such as words or to mentally break complex systems down into their component parts, but McCabe argues that these abstractions and deconstructions can't wholly capture nature's intricacy and dynamism. Instead, she suggests that humans have evolved to directly perceive meaningful structural information without our conscious awareness.

The book provides several examples of this concept, among them the remarkable process by which we recognize a human face. Rather than relying exclusively on singular features like a nose or mouth to recognize a face, we use the face's layout, or spacing between features, to drive much of the recognition process. McCabe dedicates an entire chapter to face recognition and the better part of another to showing that the same principles apply when we recognize bodies and bodily motion. Although she presents them as representative examples of how the brain works, face and body recognition are actually exceptions rather than the rule. Humans are far more sensitive to structural information in faces and bodies than other types of objects (Farah et al., 1998; Reed et al., 2003).

According to McCabe, catastrophes occur when people construct theories and rely on their internal representations of the world rather than their direct perception of it. Throughout the book, she pits theory against perception and shows how the former falls flat. Yet it is ultimately this false dichotomy that falls flat. The human ability for abstract mental representation is a crucial component of our species' success and technological advancement. It is impossible to launch a rocket, place a satellite in orbit, or construct a functioning cell phone based on perception alone. Moreover, perception and theory are far more intertwined than the author admits. No perception is truly direct. Like everything else that takes place by way of the brain, perception is a product of neural representation and is subject to influence by theory-based phenomena like attention and bias. At the same time, any theory worth a grain of salt is constructed and updated based upon information originally collected through the senses. At one point, McCabe defines theory as “ideas about the world that originate in someone's mind, rather than from observable evidence.” Yet if every reference to theory is confined to that narrow definition, the book spends 250 pages railing against a straw man. Would anyone in his or her right mind argue that decisions are best made when we close our eyes, cover our ears, and ignore all available evidence?

Many of the neuroscience descriptions in the book were also misleading or misinformed. For example, on page 91 the author argues that the brain is fractal. “Because molecules have a specific shape that can recognize other shapes, they are the ultimate pattern recognizers. Although research is needed to see how far their recognition of shapes can go, it would not be surprising to find that this molecular recognition translates up to the distributed neural networks that underlie the perception of biological motion and recognition.” This description may sound nice but it is nonsensical. Molecular signaling within and between neurons is wholly different in nature and structure from neural networks, which in turn do not necessarily give rise to structural motion processing by virtue of their interconnected wiring. Among other missteps, McCabe mixes up axons and dendrites (page 90), provides an erroneous explanation for phantom limb syndrome (page 140), and states that our perceptual skills are located in the right hemisphere (page 171).

Despite these problems, I was most troubled by the practicable message that casual readers may take away from the book. Its central argument is that theories only muddle the truth we subconsciously receive through our senses. To grasp that truth, we should follow our intuitions. As McCabe writes on page 47: “Intuition is simply the act of directly detecting structural information on a subliminal level.” Yet intuitions reflect far more than our perception of structural information. They reflect prejudice, bodily sensations, and emotions that can spur us to make bad decisions (e.g., Bolte et al., 2003; Dunn et al., 2010).

Unfortunately, the problems with this book are not unique among popular psychology books. Psychologists and neuroscientists have highlighted similar issues in books by the likes of Malcolm Gladwell and Jonah Lehrer (Engber, 2007; Pinker, 2009; Chabris, 2013). To some degree, these problems may stem from the challenges of writing about science for a general audience. They may also reflect selection or editorial pressures from publishing houses that have found a recipe for financial success in books that make sweeping, counterintuitive claims about human behavior. While more measured science books tend not to top the best sellers lists, many manage to delight and inform readers without overstating their case.

McCabe has crafted a highly readable book about human nature filled with interesting anecdotes and compelling prose. A casual reader may come away from its pages feeling that he or she learned something profound about the human mind. Yet as the book itself states, “words easily distort, reframe, or replace what actually is the case without anyone becoming the wiser.” In the end, *Coming to Our Senses* falls prey to many of the errors that it tries to warn against.

## Conflict of interest statement

The author declares that the research was conducted in the absence of any commercial or financial relationships that could be construed as a potential conflict of interest.
